# P-582. Long-term effectiveness and tolerability of Dolutegravir/Lamivudine in Korea: 3 year follow up

**DOI:** 10.1093/ofid/ofae631.780

**Published:** 2025-01-29

**Authors:** Jae Eun Seong, Sangmin Ahn, Min Han, Yongseop Lee, Jung Ah Lee, Jung Ho Kim, Jin Young Ahn, Su Jin Jeong, Nam Su Ku, Jun Yong Choi, Joon-sup Yeom

**Affiliations:** Division of Infectious Diseases, Department of Internal Medicine and AIDS Research Institute, Yonsei University College of Medicine, Seoul, Seoul-t'ukpyolsi, Republic of Korea; Yonsei University College of Medicine, seoul, Seoul-t'ukpyolsi, Republic of Korea; Yonsei University School of Medicine, Seoul, Seoul-t'ukpyolsi, Republic of Korea; Division of Infectious Diseases, Department of Internal Medicine and AIDS Research Institute, Yonsei University College of Medicine, Seoul, Seoul-t'ukpyolsi, Republic of Korea; Yonsei University College of Medicine, seoul, Seoul-t'ukpyolsi, Republic of Korea; Yonsei University College of Medicine, seoul, Seoul-t'ukpyolsi, Republic of Korea; Yonsei University College of Medicine, seoul, Seoul-t'ukpyolsi, Republic of Korea; Yonsei University College of Medicine, seoul, Seoul-t'ukpyolsi, Republic of Korea; Division of Infectious Diseases, Department of Internal Medicine, Yonsei University College of Medicine, Seoul, Seoul-t'ukpyolsi, Republic of Korea; Yonsei University College of Medicine, seoul, Seoul-t'ukpyolsi, Republic of Korea; Division of Infectious Diseases, Department of Internal Medicine, Yonsei University College of Medicine, Seoul, Seoul-t'ukpyolsi, Republic of Korea

## Abstract

**Background:**

Many studies have revealed the real-world effectiveness and tolerability of Dolutegravir/Lamivudine (DTG/3TC). However, most studies were conducted in Western countries, and data from Asian countries are still lacking; in particular, the results from long-term use have not been reported in Asian countries. As DTG/3TC has been introduced and is becoming increasingly widespread in Asia, it is necessary to investigate the maintenance rate, reasons for discontinuation, long-term effectiveness, and metabolic parameter changes.

**Figure:**
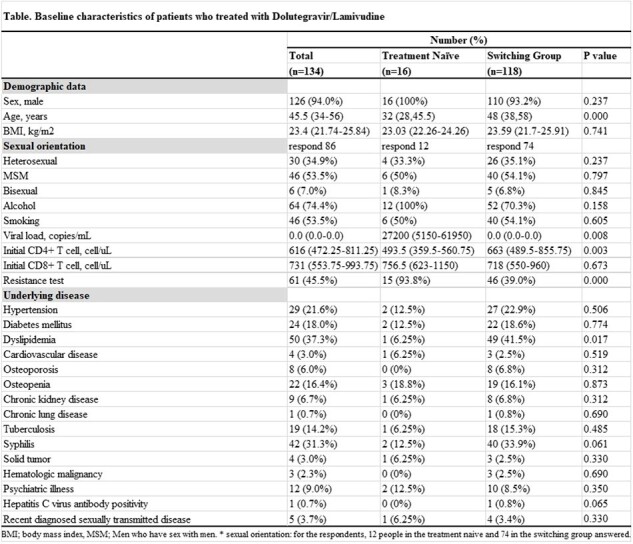

**Methods:**

We performed a retrospective cohort study with adult people with HIV treated with DTG/3TC specific combination at a tertiary hospital in Korea. Among them, those who were followed up for more than 36 months were included. Both treatment-naïve and those who switched from other antiretroviral therapies to DTG/3TC were included. Baseline characteristics, DTG/3TC maintenance rates, effectiveness, and metabolic parameters changes over 36 months were investigated.

**Figure:**
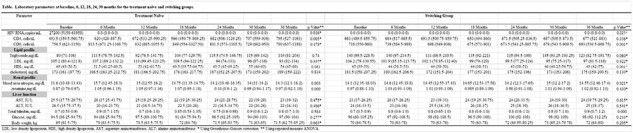

**Results:**

From July 2020 to April 2024, 305 people with HIV started DTG/3TC. Among them, 134 people with HIV followed for more than 36 months were included (16 treatment-naïve and 118 switching). Most were male (N=126, 94%), and the median age was 45.5 years. There were no cases of switching from DTG/3TC to other ARTs during the 36-month follow-up after starting DTG/3TC. All of the treatment-naïve group maintained HIV RNA < 50 copies/mL from 6 to 36 months after starting DTG/3TC. The rate of HIV RNA < 50 copies/mL at the 36 months in the switching group was 99.2% (117/118). In the treatment-naïve group, CD4 cell count increased from a median of 494 cells/uL at baseline to 795 cells/uL at 36 months (p=0.005). During the 36-month follow-up, there were no significant changes in lipid profile and body weight in both treatment-naïve and switching groups except for an increase in HDL cholesterol in the switching group (from median 45 mg/dL at baseline to median 49 mg/dL at 36-month, p=0.001).

Effectiveness of Dolutegravir/Lamivudine for the treatments-naive and switching groups

**Figure:**
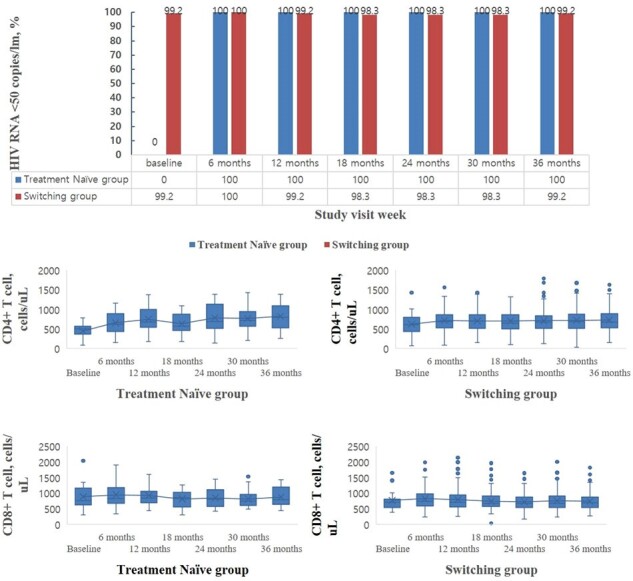

**Conclusion:**

In our long-term follow-up study conducted in South Korea, DTG/3TC worked effectively in viral suppression and CD4 count maintenance, and no significant adverse effects were observed on lipid profiles and body weight gain in both treatment-naïve and switching groups.

Tolerability of Dolutegravir/Lamivudine for the treatments-naive and switching groups

**Figure:**
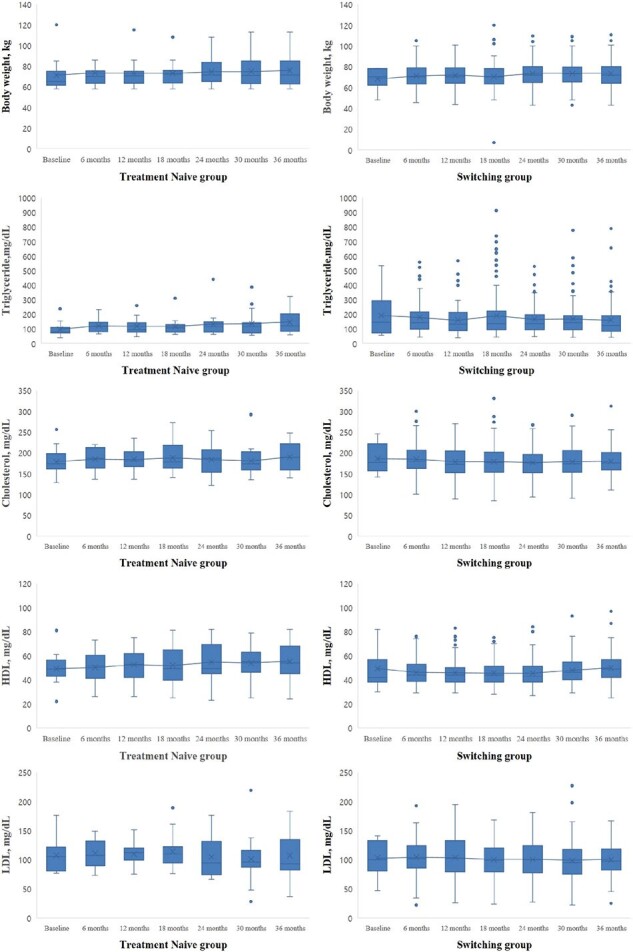

**Disclosures:**

**All Authors**: No reported disclosures

